# Assessment of physical activity and cognitive function and their potential correlation in convalescent patients of cerebrovascular disease

**DOI:** 10.1038/s41598-019-40460-6

**Published:** 2019-03-07

**Authors:** Maki Kojima, Akinori Nagano

**Affiliations:** 1 Department of Rehabilitation Medicine, Nishiyamato Rehabilitation Hospital, 3-2-2 Sasayuri-dai, Kanmaki-cho, Kitakatsuragi-gun, Nara 639-0218 Japan; 2 Graduate School of Sport and Health Science, Ritsumeikan University, 1-1-1 Nojihigashi, Kusatsu-shi, Siga 525-8577 Japan; 3 Faculty of Sport and Health Science, Ritsumeikan University, 1-1-1 Nojihigashi, Kusatsu-shi, Siga 525-8577 Japan

## Abstract

Physical activity (PA) is known to influence cognitive function. However, the impact of PA on patients with cerebrovascular disease (CVD) has not yet been elucidated. PA and cognitive function have not been measured simultaneously over time, which makes it difficult to evaluate their relationship. The purpose of this study was to investigate the change in the amount of PA recorded by ActiGraph GT3X-BT and six test scores of cognitive function, and the relationship between them in 15 patients with CVD (six women and nine men; 78.0 ± 11.6 years old). Results showed an increase in the amount of PA and scores of cognitive tests, and a significant decrease in the duration of sedentary behavior during the four months (*p* < 0.05). There were significant correlations between PA Energy Expenditure (PAEE) and Raven’s Colored Progressive Matrices test (RCPM) (*r* = 0.536, *p* < 0.0001). There were significant correlations between PAEE and Symbol Digit Modalities Test (SDMT) (*r* = 0.271, *p* = 0.036). The*r*e were significant correlations between sedentary behavior and RCPM (*r* = −0.606, *p* < 0.0001). There were significant correlations between sedentary behavior and SDMT (*r* = −0.355, *p* = 0.005). There were significant correlations between Light PA (LPA) and RCPM (*r* = 0.603, *p* < 0.0001). There were significant correlations between LPA and SDMT (*r* = 0.362, *p* = 0.005).

## Introduction

Approximately, 5.5 million people worldwide die each year from either primary insult or secondary complications of stroke^1^. Cerebrovascular Disease (CVD), including stroke (cerebral hemorrhage or cerebral infarction), damages the cerebral tissue. As a result, various forms of neurological symptoms occur in motor and cognitive functions. Concerning the value of physical activity (PA) in patients with CVD, previous studies showed that accelerometers yield valid and reliable data about the PA of patients with stroke^2^, and that the PA of community dwelling stroke survivors is low^3^. However, these studies do not show the quantitative change in PA over time.

Regarding cognitive function, cognitive decline resulting from higher brain dysfunction has multiple domains: aphasia, apraxia, agnosia, and unilateral spatial neglect^4,5^. Moreover, impairments in executive function, attention, and memory (short- and long-term) have been found in 43–78% of individuals with acute stroke, depending on the type, duration, and location of injury^6,7^. In order to improve these symptoms, cognitive rehabilitation is started on the onset of CVD^8^. Inpatient rehabilitation is typically initiated when the patient is medically stable enough to be transferred out of acute care. Typically, they are admitted to a dedicated interdisciplinary rehabilitation unit^9^. There is a wide variability in the length, type, and intensity of services provided in such programs. Kinsella and Ford^10^ found that the greatest recovery in cognitive function is expected within the first three months. In addition, Desmond *et al*.^11^ found that long-term improvement in general cognitive function might be evident after stroke.

Cognition is important for recovery in other neurological domains: patients with higher cognitive status during admission to rehabilitation had better functional outcomes, even when relevant confounding was controlled^12^. Cognitive impairments can reduce a person’s ability to understand task instructions, plan and initiate self-directed activities, and solve problems^4,13^. Therefore, further research is necessary in order to improve cognitive function with evidence-based strategies, although there are many potential rehabilitation strategies.

Currently, a growing body of evidence suggests that PA and exercise influence cognitive function. Kirk-Sanchez and McGough ^14^ reported in the review that evidence from both animal and human studies supports the role of physical exercise in modifying metabolic, structural, and functional dimensions of the brain; and in preserving cognitive performance in older adults. Barnes *et al*.^15^ reported that exercise might be one strategy to prevent or delay cognitive decline. In the meta-analysis by Chang *et al*.^16^, it was reported that the effects of acute exercise on cognitive performance were generally small. However, larger effects were possible for particular cognitive outcomes and when specific exercise parameters were used. In targeting CVD patients with cognitive decline, the relationship between PA and cognitive function has still not been elucidated. Previous clinical studies of CVD patients reported that acute treadmill exercise improves the function of hemiplegic upper extremity but not cognitive performance^17^. It has been reported that aerobic exercise improved mobility and selected cognitive domains related to motor learning, which enhances sensorimotor control after stroke^18^. In summary, regardless of the fact that there is some evidence showing that increased PA after stroke enhances cognitive performance, the pool of studies identified is small, and methodological shortcomings have been identified^19^. If PA and exercise was related to the cognitive function of patients with CVD, it would be possible to design effective interventions with PA for everyday life and cognitive rehabilitation. Therefore, it is necessary to measure PA and cognitive function simultaneously over time; and to investigate the relationship between them in patients with CVD.

Regarding the measurement of PA level, there are subjective evaluations that use questionnaires and activity record surveys^20^, and objective evaluations that use accelerometers, pedometers, global positioning systems, and other motion capture devices^21^. Subjective evaluations have found that memories of activity time and action can be inaccurate and that subjective assessment of activity intensity can vary individually. In objective evaluation, it can be difficult to decide the kind of PA that was specifically performed in the daytime while wearing the monitor, but not while sleeping. On the other hand, cognitive function is, in general, measured by cognitive testing^11^, although it is sometimes difficult to conduct among people with a damaged ability of comprehension as a result of CVD, among other issues.

The purpose of this study was to investigate the influence of PA on cognitive function in patients with CVD. We investigated the amount of PA and physical inactivity of patients with convalescent CVD over time, and investigated the potential relationship between PA and cognitive function. In our previous study, we evaluated the total energy expenditure as the amount of PA in CVD patients by using the factorial method. We found a significant correlation between total energy expenditure and everyday cognitive function^22^. In the current study, we identified PA using a wearable accelerometer, ActiGraph. ActiGraph is widely used in clinical settings and home situations. We identified the type of PA that was specifically performed in this study, since the daily activities of patients were observed and determined during 24 hours of hospitalization. This device allows long-term assessment of the patient’s body movements by means of a small solid-state recorder^23^. In clinical settings, it has been used for sleep medicine^24^, in treatment studies conducted on patients with Parkinson’s disease^25^, among patients with Chronic Obstructive Pulmonary Disease (COPD)^26^, and for stroke patients^27^. Although hospitalized stroke patients exhibit almost sedentary behaviors, ActiGraph can measure fine movements without burdening the patients. As measurement of cognitive function, we adopted six cognitive tests, which assessed attention, memory, executive function, and general intelligence as comprehensive functions, which can be conducted without difficulties in language comprehension. We hypothesized that there is a relationship between PA and cognitive function in convalescent CVD patients.

## Results

### Characteristics of subjects

The characteristics of the subjects are listed in Table 1. There were six women and nine men. Their mean age was 78.0 ± 11.6 years (range 48–94 years). Of the 15 subjects, 12 were ischemic, two were hemorrhagic, and one subject had encephalopathy. We recruited one subject with encephalopathy, since we set the inclusion criteria as wide range of brain injury. The mean value of BMI was 20.7 ± 2.7 kg/m^2^ (range 16.8–25.1), the mean value of years of education was 11.9 ± 3.0 years (range 8–16), the mean value of days since CVD was 44.3 ± 12.6 years (range 19–66), and the mean value of National Institutes of Health Stroke Scale (NIHSS) was 3.3 ± 3.2 points (range 1–12). All the subjects had experienced CVD for the first time.

**Table 1:** Individual demographic and CVD information. BMI = Body Mass Index; CVD = Cerebral Vascular Disease; NIHSS = National Institute of Health Stroke Scale.

Subject	Age (years)	Sex(male/female)	BMI (kg/m2)	Years of education	CVD type	Affected hemisphere	Days since CVD	NIHSS
1	68	M	20.2	16	Ischemic	Right	56	7
2	83	M	22.2	10	Ischemic	Left	48	12
3	77	F	22.3	15	Ischemic	Left	54	7
4	88	M	17.7	10	Ischemic	Left	36	1
5	88	F	19.4	8	Ischemic	Right	46	2
6	48	M	18.8	15	encephalopathy	Both	66	1
7	69	F	24.8	16	Ischemic	Left	55	1
8	77	M	19.9	12	Ischemic	Right	47	1
9	89	M	22.9	8	Ischemic	Left	19	1
10	74	M	16.8	12	Ischemic	Left	39	2
11	74	M	24	16	Ischemic	Left	28	1
12	67	F	20.3	14	Ischemic	Right	30	6
13	83	F	17.1	9	Hemorrhage	Right	32	3
14	94	M	18.4	8	Hemorrhage	Right	56	1
15	91	F	25.1	10	Ischemic	Right	52	4

PA of all the subjects was measured. All the subjects completed the six cognitive tests. The mean raw scores of each physical activity measure over four months are presented in Table 2. The mean raw scores of each of the cognitive assessments over four months are presented in Table 3.

**Table 2:** The mean, SD and range of the raw scores of the Physical activity measures.

Assessment	Baseline	2nd month	3rd month	4th month	
	Mean ± SD Range Median	Mean ± SD Range Median	Mean ± SD Range Median	Mean ± SD Range Median	
Physical activity energy expenditure (kcal/day)	43.6 ± 33.5 2.9–120.7 35.9	57.0 ± 49.3 4.1–184.0 41.4	55.7 ± 53.7 4.0–213.8 31.2	61.2 ± 41.6 4.8–137.4 49.6	^*^
sedentary behaviour (min/day)	1311.5 ± 79.7 1131.0–1423.6 1324.9	1280.2 ± 93.2 1017.1–1416.0 1287.5	1282.3 ± 89.5 1011.7–1418.0 1290.8	1259.6 ± 75.3 1113.0–1412.1 1256.7	^*^
Light physical activity (min/day)	125.6 ± 78.3 15.8–302.0 113.0	156.9 ± 91.9 24.0–419.0 151.2	155.3 ± 87.4 22.0–420.9 147.5	175.5 ± 73.3 27.1–324.0 182.0	^*^
Moderate physical activity (min/day)	2.9 ± 3.2 0.0–12.8 2.0	2.8 ± 3.6 0.0–12.4 1.3	2.4 ± 3.2 0.0–11.8 1.0	4.9 ± 6.6 0.0–22.2 2.0	ns
Vigorous (min/day)	0.0 ± 0.2 0.0–0.7 0.0	0.0 ± 0.0 0.0–0.0 0.0	0.0 ± 0.0 0.0–0.0 0.0	0.0 ± 0.0 0.0–0.0 0.0	ns
Very Vigorous (min/day)	0.0 ± 0.0 0.0–0.0 0.0	0.0 ± 0.0 0.0–0.0 0.0	0.0 ± 0.0 0.0–0.0 0.0	0.0 ± 0.0 0.0–0.0 0.0	ns
MVPA (min/day)	2.9 ± 3.2 0.0–12.8 2.0	2.8 ± 3.6 0.0–12.4 1.3	2.4 ± 3.2 0.0–11.8 1.0	4.9 ± 6.6 0.0–22.2 2.0	ns
Vector Magnitude (count/minute)	131.0 ± 83.1 18.6–336.6 122.1	146.8 ± 86.4 24.6–364.9 138.8	153.4 ± 78.9 38.5–371.3 145.7	163.6 ± 67.3 40.9–276.7 156.1	ns
Steps (count/miinute)	3.9 ± 2.1 1.1–8.6 3.5	4.8 ± 2.7 0.3–10.4 4.4	4.8 ± 2.7 0.3–11.8 4.3	5.2 ± 1.9 2.2–8.0 4.8	ns

^*^Friedman test p < 0.05; ns: non-significant.

**Table 3:** The mean, SD and range of raw scores of the Neuropsychological assessments.

Assessment (full score)	Baseline	2nd month	3rd month	4th month	
	Mean ± SD Range Median	Mean ± SD Range Median	Mean ± SD Range Median	Mean ± SD Range Median	
RCPM (36)	26.2 ± 5.0 13.0–34.0 26.0	27.6 ± 4.5 17.0–36.0 28.0	28.7 ± 4.3 17.0–34.0 29.0	28.4 ± 5.8 12.0–35.0 29.0	^*^
SDMT	21.4 ± 10.4 1.0–49.0 22.0	23.6 ± 9.6 9.0–44.0 22.0	25.3 ± 10.8 8.0–51.0 23.0	26.3 ± 10.4 12.0–53.0 23.0	^*^
Symbol Trails (10)	7.0 ± 3.1 0.0–10.0 8.0	8.7 ± 1.8 4.0–10.0 10.0	7.9 ± 2.8 0.0–10.0 8.0	8.3 ± 2.0 5.0–10.0 10.0	ns
Symbol Cancellation (12)	11.1 ± 3.0 0.0–12.0 12.0	11.8 ± 0.4 11.0–12.0 12.0	11.8 ± 0.4 11.0–12.0 12.0	11.8 ± 0.4 11.0–12.0 12.0	ns
Maze (8)	6.6 ± 1.6 3.0–8.0 7.0	6.4 ± 1.6 3.0–8.0 7.0	6.1 ± 1.9 2.0–8.0 6.0	6.4 ± 2.2 0.0–8.0 7.0	ns
Design Memory (6)	4.5 ± 1.2 2.0–6.0 5.0	5.1 ± 1.0 3.0–6.0 5.0	5.1 ± 0.9 3.0–6.0 5.0	4.9 ± 1.1 3.0–6.0 5.0	ns

^*^Friedman test p < 0.05; ns: non-significant.

### Change in physical activity over four months

**Figure 1:**
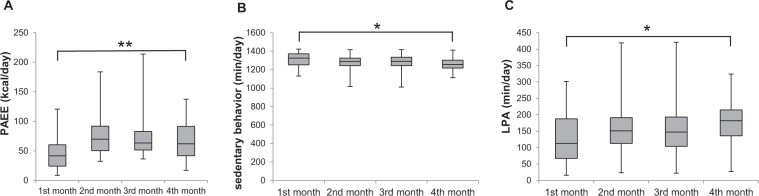
Box plots showing change in the value of physical activity over four months. (**A**) PAEE, (**B**) Sedentary behavior, and (**C**) LPA. The boxes extend from the 25th to the 75th percentile, with a line at the median and error bars defining the highest and lowest values. Friedman test was performed followed by Wilcoxon post hoc test, comparing the value of each month (*p < 0.05 and **p < 0.01; Bonferroni-adjusted comparisons).

We investigated the change in 9 parameters of PA over four months. Significant changes were seen as shown in Fig. 1 over the four months. The value of PAEE and LPA significantly increased, and the value of sedentary behavior significantly decreased over four months. There was a significant main effect of time points (the first month through the fourth month) in Friedman Tests, regarding PAEE (χ^2^(3) = 10.760, *p* = 0.013, Fig. 1A), sedentary behavior (χ^2^(3) = 13.640, *p* = 0.003, Fig. 1B), and LPA (χ^2^(3) = 11.000, *p* = 0.012, Fig. 1C). We conducted Wilcoxon ‘s post hoc test in order to investigate the differences among the 1st, 2nd, 3rd and 4th months. There were significant differences in PAEE, sedentary behavior, and LPA between the 1st month and the 4th month, according to Wilcoxon post-hoc test (Bonferroni-adjusted comparisons), that is, PAEE (z = −3.181, *p* = 0.006, Fig. 1A), sedentary behavior (z = −2.897, *p* = 0.024, Fig. 1B), and LPA (z = −2.783, *p* = 0.03, Fig. 1C). There were no differences between any of the other time points for any of the PA variables.

### Change in cognitive function over four months

**Figure 2:**
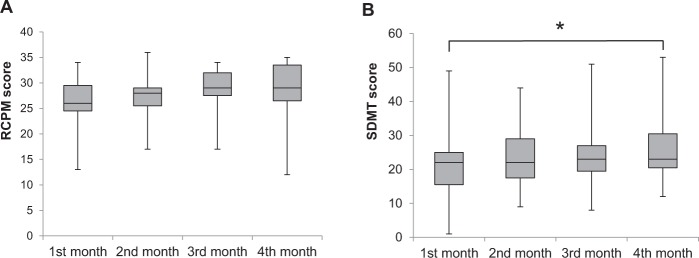
Box plots showing change in the value of cognitive function over four months. (**A**) RCPM, (**B**) SDMT. The boxes extend from the 25th to the 75th percentile, with a line at the median and error bars defining the highest and lowest values. Friedman test was performed followed by Wilcoxon post hoc test, comparing the value of each month (*p < 0.05; Bonferroni-adjusted comparisons).

We investigated the change in 6 tests of cognitive function over four months. Significant changes were seen as shown in Fig. 2 in the 15 subjects over the four months. The value of RCPM, SDMT significantly increased over four months. There was a significant main effect of time points (first month through fourth month) in Friedman Tests, regarding RCPM (χ^2^(3) = 8.2, *p* = 0.042, Fig. 2A), SDMT (χ^2^(3) = 9.875, *p* = 0.020, Fig. 2B). We conducted Wilcoxon ‘s post hoc test in order to investigate the differences among 1st, 2nd, 3rd and 4th months. There were significant differences in RCPM and SDMT, according to Wilcoxon post-hoc test (Bonferroni-adjusted comparisons), between the 1st and 4th month (z = −2.649, *p* = 0.048, Fig. 2B). There were no differences between any of the other time points for any of the cognitive variables.

### Relationship between physical activity and cognitive function

**Figure 3:**
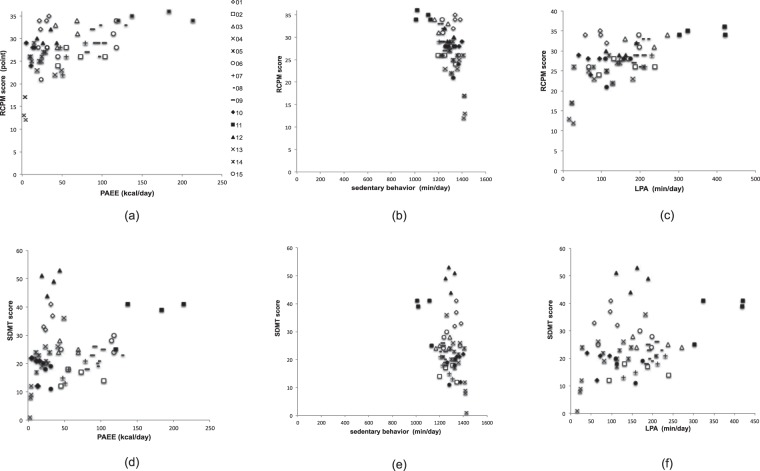
Relationship between the value of PA and cognitive function. The relationship between the score of PA for each month and the score of cognitive function for each month. Data for 15 individual subjects are presented. Each marker corresponds to an individual subject, and is commonly used in plots (**a**) through (**f**).

Figure 3(a–f) shows the relationship between the score of PA for each month and the score of cognitive function for each month for 15 subjects.

There were significant correlations between the mean value of PAEE and the mean value of RCPM (*r* = 0.536, *p* < 0.0001), that of sedentary behavior and that of RCPM (*r* = −0.606, *p* < 0.0001), that of LPA and that of RCPM (*r* = 0.603, *p* < 0.0001), that of PAEE and that of SDMT (*r* = 0.271, *p* = 0.036), that of sedentary behavior and that of SDMT (*r* = −0.355, *p* = 0.005), and that of LPA and that of SDMT (*r* = 0.362, *p* = 0.005).

There were significant correlations between the mean value of PAEE and the 4th month’s score of RCPM (*r* = 0.699, *p* = 0.004), the mean value of PAEE and the 4th month’s score of SDMT (*r* = 0.481, *p* = 0.070), the mean value of sedentary behavior and the 4th month’s score of RCPM (*r* = −0.744, *p* = 0.001), the mean value of sedentary behavior and the 4th month’s score of SDMT (*r* = −0.544, *p* = 0.036), the mean value of LPA and the 4th month’s score of RCPM (*r* = 0.735, *p* = 0.002), and the mean value of LPA and the 4th month’s score of SDMT (*r* = 0.588, *p* = 0.021).

## Discussion

In the current study, we examined PA and cognitive function for four months, and the relationship between them among 15 CVD patients in convalescent hospital wards. We first evaluated the change in PA by collecting data from the ActiGraph which was worn by subjects, and from cognitive function item scores of six assessments over four months from the time of admission. Next, we examined the correlation between the value of PA measures and cognitive function item scores.

As shown in Figs 1 and 2, significant changes in the PA and cognitive function were found. In previous studies, Manns and Evan ^28^ reported that minutes of activity and length of activity bouts significantly increased from predischarge to 6 weeks postsdischarge. Although the term of measurement was different from that study, we obtained a similar result. LPA increased significantly, but higher intensity activity measures, such as MVPA did not, and patients gradually engaged less in sedentary behaviors. Regarding cognitive function, RCPM and SDMT scores increased significantly, but other assessments did not. It was indicated that general intelligence, and divided attention in many domains of cognitive function improve in CVD patients in this study. Hochstenbach *et al*.^29^ reported that there was room for improvement in all cognitive domains after stroke. The study showed that the largest improvement was found in the attentional domain and the least improvement was found in the memory domain. In the current study, intelligence and divided attention improved, which is consistent with this preceding study. Nonetheless, as we did not have control subjects, the cause-and-effect relation between PA and cognitive function could not be revealed in the current study. On the other hand, selective and alternated attention, executive function, and memory did not improve in four months in the current study. Particularly, the value of the symbol cancellation task was stable. A previous study^29^ showed that attention domain improved but memory domain did not improve through 2-year follow up study. We obtained controversial result compared to the previous study, and it is necessary to confirm this point recruiting greater numbers of subjects. Regarding memory, there is a possibility that memory is difficult to improve. Or, it could be considered to be a ceiling effect since the subject of the current study was able to almost completely carry out the memory task.

It was suggested that the higher the PAEE and LPA, the higher the cognitive function value; and the lower the sedentary behavior is, the higher the cognitive function value. The present findings indicate that PA among people with CVD is potentially related to their cognitive function.

The current study has several limitations. First, the current study had a small sample size comprising individuals in an inpatient rehabilitation unit. Thus, our findings are not likely to be conclusive. To intensively investigate changes in PA and the cognitive function of CVD patients over time, and to establish evidence of the positive relationship between PA and cognitive function in CVD patients, it is necessary to recruit a greater number of participants. Second, regarding cognitive assessments, the score on Symbol Cancellation, Design Memory, and Mazes did not change over the four months. It is necessary to confirm whether these domains could not actually improve or whether other tests would have been more appropriate. We will conduct a future follow up study that will consider these points.

In the current study, we found a significant change in the score of PAEE, LPA, sedentary behavior, and general intelligence and attention as cognitive functions. We also found that PA was potentially related to cognitive function among people with brain damage. Aarsland *et al*.^30^, Larson *et al*.^31^, and Podewils *et al*.^32^ reported that cognition in Alzheimer’s disease patients was also highly related to energy expenditure and exercise, and that PA helped to prevent disease progression. Likewise, the present study found a potential correlation between PA and cognitive function in patients with CVD. However, the current study did not explore a causal relationship between PA and cognitive function. Thus, in the future, it will be necessary to further investigate through intervention experiments, whether PA is effective in improving the cognitive function of patients with CVD using an intervention study. We will do intervention studies to clarify that PA is valid for cognitive function.

## Materials and Methods

### Participants

Adults who experienced cerebrovascular disease, and who were suspected to have cognitive deficit based on the Mini-Mental State Examination^33^, were recruited from Nishiyamato rehabilitation hospital (Nara, Japan). The inclusion criteria were patients who (1) had diseases and disorders of the nervous system, that is, nonspecific cerebral vascular disorders including post stroke (ischemic or hemorrhagic), traumatic brain injury, and encephalitis and encephalopathy, (2) completed their acute treatment and were in stable condition, and (3) were ready for rehabilitation. The exclusion criteria were the (1) appearance of delirium and fever symptoms during hospitalization, (2) large fluctuation in mental state, (3) refusing rehabilitation, (4) inability to stay in the hospital with other patients, and (5) inability to wear the monitoring device on the waist. Subjects in the inpatient rehabilitation unit typically underwent three basic therapies: physical therapy (PT), occupational therapy (OT), and speech language therapy (ST). PT provides fundamental training to perform basic activities, gait training, and balance training. OT provides training to perform activities of daily living (ADL), instrumental activities of daily living (IADL), and upper extremity training. ST provides training on recovering speech, language, and cognitive communication; and on swallowing. OT and ST perform the paper-and-pencil cognitive tasks in a quiet therapy room.

This study was conducted according to the Declaration of Helsinki and approval for the project was obtained from the local ethics committee (Department of Rehabilitation Medicine, Nishiyamato Rehabilitation Hospital, Nara, Japan, protocol number 16). All participants gave their written informed consent to participate in this study, which was approved by Nishiyamato rehabilitation hospital. Procedures in accordance with institutional guidelines were followed in this study.

### Procedure

**Figure 4:**
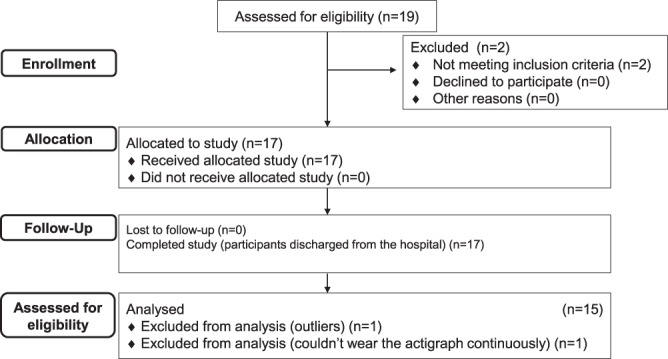
Flow chart of selection of the participants. This flowchart shows how many patients were screened, how many were ineligible, how many declined participation, and how many enrolled in this study.

The value of PA, sedentary behavior, and six cognitive test scores were collected per month from the first month of admission to the fourth month. PA and sedentary behavior were recorded for two to seven days (at least two days) in a month. Participants wore a PA monitor, ActiGraph GT3X-BT (ActiGraph Corp., Pensacola, Florida) on the waist. A speech language pathologist conducted six cognitive tests on any day of wearing an ActiGraph. This study was carried out for total for 15 months in total. The subjects screened in this study are shown in the flowchart (see Fig. 4).

First, the average values of PA per day for all subjects were calculated. As PA value, we adopted nine outcomes which were Physical Activity Energy Expenditure (PAEE), sedentary behavior, time spent on Light-intensity Physical Activity (LPA), Moderate-intensity Physical Activity (Moderate Physical Activity), Vigorous-intensity Physical Activity (Vigorous Physical Activity), Very Vigorous-intensity Physical Activity (Very Vigorous Physical Activity), time spent on Moderate-to-vigorous intensity physical Activity (MVPA), vector magnitude overall physical activity counts per minute (cpm; Vector Magnitude Count), and steps. Count per minute (cpm) represents colloquial expression for number of times something (e.g., heartbeat) happens within a 60-second duration^34^. At the same time, the score of six cognitive tests was measured, and then, these changes were noted. We identified significant changes in the values of nine outcomes of PA value and six tests scores of cognitive measurements. Next, we examined the correlation between PA value and the cognitive test scores.

### Measurement of physical activity

PA was measured with an accelerometer, ActiGraph GT3X-BT (ActiGraph Corp., Pensacola, Florida), worn on the waist. Concerning the reliability of Actigraph in people with post-stroke, the Intraclass correlation coefficients (ICC) was high when comparing activity counts for the paretic and non-paretic hips (0.96)^35^. The location of wearing an accelerometer, e.g., waist, wrist, and ankle, etc. may influence PA estimates. As a validation study, Shiroma *et al*.^36^ reported that similar patterns of waist and wrist activity accrual indicated that each location was capable of estimating total PA volume. LaMunion *et al*.^37^ also reported that Actigraph y-axis and vector magnitude counts did not level off when the monitoring device was worn at the hip. Therefore, we observed that reliable measurement of PA can be obtained by wearing Actigraph at the waist. We included the hemi-paretic patients in this study. It was considered that the device would be an obstacle, if it was worn on the non-paretic wrist and ankle, which are used actively throughout the day. Therefore, this device was worn on the waist in this study. Each participant wore the device continuously: day and night. The inclusion criteria regarding the use of accelerometers were the ability to wear the device continuously (without removing) for at least two days to a maximum of seven days. This protocol was used because some participants could not continuously wear the device for a long period of time. We used the average value per day for two to seven days in statistical analysis. The activities of the participant were determined per hour. ActiLife software (version 6.13.3; ActiGraph Corp., Pensacola, Florida) was used to initialize the accelerometers and download the data. Customized software was used for data reduction and analysis. We used vector magnitude units (VMU). VMU represents total movements in three planes for each minute, and is used to categorize PA between high and low levels. The outcome variables of PA and the thresholds we used in this study were PAEE (kcal/day), sedentary behavior (min/day;  < = 99 cpm), LPA (min/day; 100–1951 cpm), Moderate Physical Activity (min/day; 1952–5274 cpm), Vigorous Physical Activity (min/day; 5725–9498), Very Vigorous Physical Activity (min/day > = 9499), MVPA (min/day; > = 1952 cpm), Vector Magnitude Count (counts/minute), and steps (counts/minute) which is obtained from the algorithms of the Freedson Combination^38^. We used the Low-Frequency Extension filter. The Low-Frequency Extension option should only be used in very specific cases where PA is at such a low level that it might otherwise be eliminated with the normal filter. We used this filtering because subjects of this study exhibited very slow shuffling movements. The epoch length was 60 seconds, which represent the amount of time the raw acceleration data is summed after the filter is applied.

### Measurement of cognitive function

We conducted six cognitive tests in this study: Raven’s Progressive Matrices Test (RCPM); Symbol Digit Modalities Test (SDMT); and four tests from the nonlinguistic tasks of Cognitive Linguistic Quick Test^39^, including Symbol Trails, Symbol Cancellation, Mazes, and Design Memory. These tests were conducted because they can assess different main aspects of cognitive function, and can be administered easily; even participants with language comprehension difficulties could understand and conduct these tests. Participants of this study could use pencils for writing using their dominant or non-dominant hand.

### Raven’s colored progressive matrices test

This test assesses general intelligence. Raven’s Progressive Matrices (Raven, 1976) provide a trusted, nonverbal assessment of intelligence. Since these scales minimize the impact of language skills and cultural bias, they are particularly well suited for measuring the intelligence of individuals with reading problems or hearing impairment, as well as those whose native language is not English. Appropriate for both children and adults, Raven’s Progressive Matrices measure two complementary components of general intelligence: the capacity to think clearly and make sense of complex data (educative ability), and to store and reproduce information (reproductive ability)^40,41^.

### Symbol digit modalities test

This test assesses divided attention. The SDMT (Smith, 1982)^42^ was developed to identify individuals with neurological impairment. The SDMT assesses key neurocognitive functions that underlie many substitution tasks, including attention, visual scanning, and motor speed. The SDMT requires individuals to identify nine different symbols corresponding to the numbers one through nine, and practice writing the correct number under the corresponding symbol. Afterwards, they manually fill the blank space under each symbol with the corresponding number. The second oral administration is then completed. The participant is given a blank copy of the test and asked to state the correct number for each corresponding symbol. They have 90 seconds to complete each of these administrations. The written and oral score is calculated by totaling the number of correct answers for each section. Oral and written administrations provide two different indices of functioning, which assess attention, scanning abilities, and motor skills^43^.

### Symbol trails

This test assesses the executive functions of planning, working memory, and mental flexibility without placing demands on the language system. However, this task also calls upon visual attention and perception. Two learning trails involving the same concepts of size and shape are used in preparation of the test item, which involves drawing a single line to connect a total of 11 circles and triangles in an alternating fashion according to size and shape and beginning with the smallest circle. The maximum possible score is 10 points^39^.

### Symbol cancellation

This test assesses visual attention, scanning, discrimination, inhibition, and response shifting within four quadrants of space. Thirty-six abstract symbols are arranged in a pseudo-random fashion with the target stimulus appearing thrice in each quadrant of space to allow for assessment of visual field deficits and visual neglect. Discriminating the target stimulus from resembling symbols is necessary. The task is to cross out target symbols. The highest possible score is 12 points^39^.

### Mazes

This test assesses executive functions, specifically those involved in planning a course of action, rejecting/inhibiting incorrect choices, and correcting mistakes. Two mazes of different levels of difficulty are used. The highest possible score for each maze is four (correct solution) with a total of eight points^39^.

### Design memory

This test assesses immediate/working visual memory and attention without language demands. Two target abstract designs are presented one at a time for memorization. They must be identified immediately from arrays of six designs that include four foils that are similar to the targets. The highest possible score is six points^39^.

### Statistical analysis

Due to the small sample size, non-parametric statistics were used. Friedman Tests were performed to compare the values per month from the first month of admission to the fourth month: average kcal values per month for PAEE, sedentary behavior time, time spent on LPA, Moderate-intensity Physical Activity, Vigorous-intensity Physical Activity, Very Vigorous-intensity Physical Activity, and MVPA, vector magnitude overall physical activity counts per minute, step counts per minute, and six cognitive test scores including RCPM, SDMT, Symbol Trails, Symbol Cancellation, Mazes, and Design Memory. When significant differences were found in Friedman Tests, Wilcoxon Tests as post-hoc test were conducted to examine the differences between each value by using Bonferroni-adjusted correction. For the parameters in which significant differences were found in Friedman Tests, the relationship between the mean value of PA for all four months and the mean value of cognitive tests score for all four months, and the relationship between the mean value of PA and the cognitive tests score of the 4th month were analyzed using Spearman’s correlation coefficient. The level of statistical significance was determined as *p* = 0.05. For the statistical analysis, we used IBM SPSS statistics for Mac, version 25.
